# The Heterogeneous Effects of Different Environmental Policy Instruments on Green Technology Innovation

**DOI:** 10.3390/ijerph16234660

**Published:** 2019-11-22

**Authors:** Ming Yi, Xiaomeng Fang, Le Wen, Fengtao Guang, Yao Zhang

**Affiliations:** 1 School of Economics and Management, China University of Geosciences, Wuhan 430074, China; yiming@cug.edu.cn (M.Y.); zhangyao@cug.edu.cn (Y.Z.); 2 Energy Centre, The University of Auckland, Auckland 1142, New Zealand; l.wen@auckland.ac.nz; 3 School of Economics and Management, North China Electric Power University, Beijing 102206, China; guangft@ncepu.edu.cn

**Keywords:** green technology innovation, environmental policy instruments, panel threshold estimation, regional heterogeneity

## Abstract

Environmental regulation is an important driving force of green technology innovation. In this paper, environmental policy instruments are classified into three categories: command-control, market-incentive and social-will. Based on the panel data of 30 provinces in China from 2010 to 2017, a fixed effect model and a panel threshold regression model are used to test the heterogeneous effects of different types of environmental policy instruments on the green technology innovation in China. The results show that: (1) Overall, China’s environmental policy instruments do not provide sufficient impetus for green technology innovation; (2) The impact of command-control environmental policy instruments on green technology innovation has a single threshold effect. When its intensity exceeds a certain threshold, green technology innovation is improved. The impact of market-incentive environmental policy instruments on the green technology innovation shows a double threshold effect, that is to say, only when its intensity maintained within a reasonable interval, can green technology innovation be promoted by it; (3) There is significant spatial difference in the impact of different types of environmental policy instruments on green technology innovation. In order to induce green technology innovation, it is necessary to formulate a combined and differentiated environmental policy system, while rationally adjusting the strength of different types of environmental policy instruments.

## 1. Introduction

Green development is the general trend of international development. At present, the pressure of international climate negotiations such as the Kyoto Protocol and the Paris Agreement will continue, and the green race may change the country’s comparative advantage (Fankhauser et al., [1]; OECD [2]). Green technology is a general term for various technologies, processes or products aimed at improving the quality of ecological environment and promoting the intensive use of resources and energy. It is a new technology that integrates ecological environment protection with economic development, and its special attributes and values determine that green technology innovation is the only way to fundamentally solve ecological and environmental problems, and is an important technological path to promote green development. Green technology innovation is the integration of technological innovation and ecosystem, including the whole process of green technology from source research and development to achievement transformation and final marketization (Song and Wang [3]). By promoting the research and development of green technology and promoting the popularization and application of green technology, it is conducive to improving the efficiency and benefits of water conservation, energy conservation, material saving and land saving. In particular, if green technology is revolutionary, it can achieve an order of a quantitative or qualitative leap in green development and achieve a “win-win” between economic development and environmental protection.

Scholars have analyzed environmental issues from different angles such as Pigou’s “externality”, Coase’s “transaction cost”, Arrow’s “information asymmetry”, North’s “free rider”, etc., all of which put forward new perspectives, but didn’t pay attention to the dynamic role of technology. As for the relationship between environmental protection and economic growth, the pioneering research by Grossman et al. found that there was an inverted U-shaped curve between pollution level and per capita income level [4], and the environmental Kuznets curve (EKC) hypothesis was subsequently verified and theoretically explained by a large number of empirical studies (Katircioglu [5]). On the whole, the direct reasons for the decline of pollution level with economic growth can be summarized into two categories: one is the structural effect of the shift of pollution-intensive industries from developed countries to developing countries; and the other is the effect of green technology innovation in favor of energy conservation and clean production.

Enterprises are the main drivers of regional green technology innovation. From the value chain perspective, enterprises accumulate a large amount of capital, innovative human capital, market environment and other resources to meet the whole process of green technology from research and development to production. However, under the conditions of the modern market economy, there are many problems in solving environmental problems through green technology innovation as the sole tool: First, there are no unified environmental damage measurement indicators when enterprises themselves face the problem of environmental pollution and destruction brought about by production and operation, so that the investment intensity and direction of green technology innovation can’t be determined; Second, in view of the environmental externalities and innovation externalities of green technology innovation, the benefits of green technology research and application of results may be less than their actual investment. In the absence of environmental regulations or with environmental policy instruments that are not applicable or imperfect, enterprises may lack sufficient green technology innovation power; Third, the green technology innovation is based on multiple disciplines such as technology, economy and ecology. Without the guidance of environmental policies, enterprises have a high risk of failure in green technology innovation (Huang and Zhang [6]). It can be seen that constructing a reasonable environmental policy system is necessary and important for enterprise green technology innovation. Therefore, it is necessary to systematically investigate the influence mechanism and actual effect of environmental policy instruments on enterprise green technology innovation, so as to provide reference for formulating and improving environmental policy instruments.

The research in this paper mainly answers three questions: First, is there heterogeneity effects of different environmental policy instruments on green technology innovation in China? Second, does the impact of different environmental policy instruments on green technology innovation have a threshold effect in China? Third, does the impact of different types of environmental policy instruments on green technology innovation have significant spatial differences in different regions of China? In order to answer the above questions, this paper selects green technology patents to measure enterprise green technology innovation, and divides environmental policy tools into three types: command-control, market-incentive and social-will (Iraldo et al. [7]) based on the OECD classification standards. And on the basis of revealing the impact mechanism of different types of environmental policy tools on enterprise green technology innovation, with the economic data of China’s 30 provinces in 2010–2017, the paper tests the heterogeneous impacts of different environmental policy tools on enterprise green technology innovation using the fixed effect model and panel threshold model.

The significance of this paper is to provide valuable literature foundation and research ideas for the theoretical research of green technology innovation in China by answering the above three questions, and to provide reference for the improvement of China’s environmental policy instrument system. The following contents are arranged as follows: Section 2 is the literature review; Section 3 presents the mechanism analysis of different environmental policy instruments influencing green technology innovation of enterprises; Section 4 is the research model design; Section 5 is the analysis of empirical results. Finally, the Conclusions section presents the study conclusions, and proposes the corresponding policy recommendations and necessary discussion.

## 2. Literature Review

The bias of technological progress has a profound impact on the environmental outcomes of economic activities. New technologies may either increase pollution, or reduce, or replace polluting activities. This bias is inherent in the process of economic growth (Jaffe and Peterson [8]). Therefore, in recent years, research on environmental economy and policy has increasingly introduced technological progress as an endogenous variable into an analytical model, discussing the relationship between environmental protection and technological innovation and economic growth, and gradually derived ecological innovation on this basis (Fussler and James [9]; Sanni [10]), environmental innovation (Rennings et al. [11]; Makkonen and Inkinen [12]), sustainable innovation (Melville [13]), and the concept of green technology innovation is usually associated with the above concepts. Green technology innovation is “the research, development and application of green technology, products and processes, including the whole process of green technology from the source of research and development to the transformation of achievements and final marketization”. In the past 20 years, different analytical perspectives or methods have been used to study the dynamics, characteristics, types, driving factors of green technology innovation and its impact on economic and social systems (Rennings [14]; Chen et al. [15]; Arfi et al. [16]; Wurlod and Noailly [17]).

Regarding environmental regulation policies, the most typical “Porter Hypothesis” [18] believes that appropriate environmental regulations will stimulate technological innovation, bring “innovation compensation” and competitive advantage, and scholars have subsequently carried out a large number of empirical tests of “porter hypothesis”. Due to differences in research background and analytical perspective, the conclusions are much differentiated. The views can be divided into the following three categories:

First, the “Porter Hypothesis” exists, that is, environmental regulation can promote enterprise innovation. The findings of Berman and Bui [19] and Yang et al. [20] support this view, suggesting that strengthening environmental regulation has a significant role in promoting technological innovation in enterprises, and another study by Ambec et al. [21] found that performance-based or market-based environmental regulations can promote business innovation. Second, this conclusion is contrary to the “Porter Hypothesis”, that is, environmental regulation can inhibit enterprise innovation, or, has either no significant or uncertain effect on enterprise innovation. For example, Ramanathan et al. [22] showed through theoretical research that strict environmental regulation has not significantly promoted technological innovation in enterprises, and has not brought enterprises enough benefits to make up for the cost of environmental management, but has led to a decline in corporate profits; Calel [23] found that in some cases, higher emission prices actually reduce enterprises’ innovation in emission reduction technologies; Vollebergh [24] found that the effect of environmental regulation on enterprise innovation is uncertain and certain conditions should be met. Third, the relationship between environmental regulation and enterprise innovation is non-linear. For example, Nesta et al. [25] found that the dynamic incentives of environmental policies become effective just above the median level of relative competencies of green technology innovation. Wang and Liu [26] took the data of Chinese industrial enterprises from 1998 to 2011 as a sample and used the two-stage least squares estimation method to find that the relationship between environmental regulation and TFP of enterprises conforms to the inverse N-shaped.

With the continuous enrichment of the environmental policy instruments system, many scholars adopt quantity of patent applications as the proxy variable for enterprise innovation, using fixed effect models to explore the effects of different types of environmental regulation on enterprise innovation: Downing and White [27], and Milliman and Prince [28] argue that compared to the impact of command-based environmental policy tools on green technology innovation, under the market-based system (such as emission fees or permits), the incentive effect on enterprise technology innovation is stronger. At the same time, Burtraw [29] also emphasizes that the shift from command and control methods to more flexible emission trading schemes can enhance enterprise innovation, and market-based environmental policies are found to be more productive than non-market instruments (Albrizio et al. [30]).

Enterprises’ technological innovation activities not only include general technological innovation aiming at increasing production efficiency and economic benefits, but also include green technological innovation targeted at avoiding or reducing environmental damage (Vicki [31]). Compared with the general technology innovation, green technology innovation has the dual externalities of environmental externality and innovative externality (Rennings [14]). The willingness of enterprises to increase their investment in green technology research or adopt patented green technology will be affected by multiple factors, among which, environmental regulation is an important factor in the innovation of enterprise green technology (Lanjouw and Mody [32]; Popp et al. [33]; Dekker et al. [34]; Costarmini and Mazzanti [35]). Brunnereier and Cohen [36] used the number of environment-related patent applications as a measure of green technology innovation, and found that there is a significant positive correlation between pollution treatment expenditures and enterprises’ green technological innovation. Horbach [37] pointed out that since negative externalities are the characteristics of most environmental problems and green technology innovation is less market-incentive than other innovations, governments’ environmental policy tools will be conducive to green technology innovation. Dechezleprêtre [38] confirmed that there is a positive correlation between vehicle emission standards and patent inflow and development of vehicle emission control technology. Qi et al. [39] found that since the expansion of the emissions trading policy in 2007, emissions trading has gradually become more active as one of the market-driven environmental policy tools. Moreover, compared with other patent types, emission trading has a stronger effect on green invention patents. However, some scholars believe that environmental policies may inhibit green technology innovation. For example, Wagner [40] found that the implementation level of environmental policy only positively impacts environmental process innovation, but is negatively correlated with the general patent activity level of enterprises. According to empirical tests by Jaffe and Palmer [41], even in industries with more than 40% patents in the process innovation process, there is no statistically significant relationship between pollution control expenditures and patent behavior activities under environmental regulations.

On the whole, research on the influence of environmental regulation on enterprise technological innovation has achieved fruitful results, but the existing research still needs further improvement as relevant research on the impact of environmental regulation on green technology innovation has paid insufficient attention to the differences in environmental policy instruments and the resulting heterogeneity. In fact, as a response to environmental issues, since the late 1960s, various forms of environmental policy tools such as news media propaganda, compulsory orders, punishments and subsidies have emerged. The implementation methods and implementation effects of different types of environmental policy instruments are quite different. Therefore, when measuring the impact of environmental regulation on the green technology innovation of enterprises, it should be examined differently. At the same time, attention should also be paid to the heterogeneous effects that may be caused by different policy implementation intensity; Finally, due to the different ecological and environmental conditions, types of pollution sources and government policies for green development in different regions, the impact of different types of environmental policy instruments on green technology innovation of enterprises also has spatial differences. Therefore, this problem also needs to be tested.

## 3. Influence Mechanism

**Figure 1:**
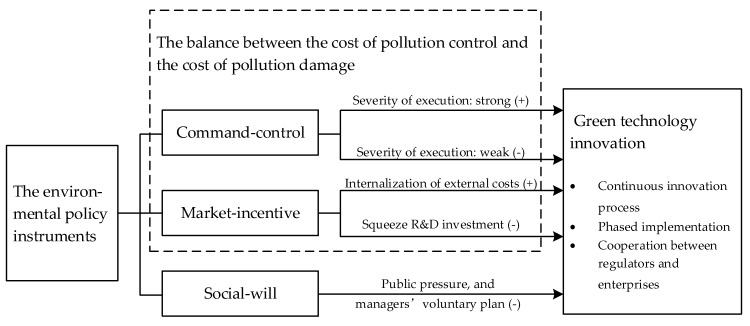
Influence mechanism of different types of environmental policy instruments on green technology innovation.

In the practice of environmental regulation, the government must first solve the problem of determining what is the best level of pollution? Under the theoretical framework of the neoclassical school, considering the effectiveness of cost allocation, the implementation goal of environmental regulation is to reach the optimal pollution level. At the optimal pollution level, the sum of pollution control costs and pollution damage costs is minimal (Tietenberg and Lewis [42]). With the environmental protection concept of low-carbon economy deeply rooted in the hearts of the people, most countries no longer only consider environmental costs, but introduce the concept of environmental cost-benefit analysis into the field of environmental regulation. Environmental cost-benefit analysis is a combination of cost-benefit analysis theory and environmental science. Essentially, it is an estimate of the input-output relationship in order to pursue utility maximization. The basic idea is that, on the basis of analyzing the economic and environmental benefits of a certain activity, the environmental benefits are monetized through certain rules and standards, and then superimposed with the economic benefits to obtain comprehensive economic benefits. According to the cost-benefit theory, relevant scholars believe that environmental regulation will impose cost constraints on enterprises and hinder their development in a short period of time. But in the long run, driven by different types of environmental policy instruments, enterprises will face a variety of cost choices, which will have a differentiated impact on their green technology innovation (Hammitt et al. [43]). Therefore, this paper attempts to construct a model of the impact mechanism of different environmental policy instruments on enterprise green technology innovation (as shown in Figure 1).

First of all, command-control environmental policy instruments are the most direct product of the government’s response to environmental pollution and destruction. At the same time, command-control environmental policy instruments including market access, product bans, technical specifications, emission performance standards, emission discharge permits, etc., are also the main form of global environmental policy. The starting point of command-control environmental policy instruments is to compulsively regulate the pollution emission behavior and standard in the production process. Due to the nature of quasi-public goods in environmental resources, there may be “crowding effect” and “overuse” problems in use. The scarcity of environmental resources makes the environment a commodity when there is a limited supply situation of overuse and damage to the environment in the process of production. For the whole society, the optimal condition for output is that the social marginal cost is equal to the social marginal benefit. When negative externalities exist, the marginal cost of enterprises will be lower than the marginal cost of society, and the supply of enterprises will increase, thus leading to a vicious circle of environmental pollution. Therefore, the government introduces command-control environmental policy instruments to force enterprises to consider pollution control in the process of production and operation through mandatory command means. Among them, when the negative external effects are small, the government’s implementation of command-and-control environmental policy tools is weak. At this time, enterprises pay more attention to how to obtain economic benefits through the increase of production to compensate for pollution control costs, or through secret emission and leakage emission to avoid environmental compensation, so that the importance of green technology innovation is neglected. In addition, the incentive of green technology innovation could flatten out with the regulation is simple achieved, and analogously to market-based mechanisms, complying with a (binding) standard comes with a certain cost, which could decrease innovation activity; When the negative environmental externalities are too large, relying solely on market will lead to the misallocation of resources and the aggravation of environmental pollution. At this time, the implementation intensity of command-control environmental policy instruments increases, and the risk of pollution damage faced by enterprises will increase. When the intensity reaches a certain level, it is impossible to make up for the excessive pollution control cost by increasing the output to gain economic benefits, thus triggering the green technology innovation of enterprises. Therefore, this paper proposes to research hypothesis 1: when the intensity of command-controlled environmental policy instruments is low, it will inhibit the green technology innovation of enterprises; when it exceeds a certain intensity, it will promote green technology innovation.

Secondly, the market-incentive environmental policy instruments are incentives for polluting enterprises that are more in line with environmental protection objectives. This is done through collecting economic compensation from polluters through environmental taxes and fees, to achieve emission reduction effects through emission trading in the reduction of emission standards and emission reduction of internal polluters. In theory, market-based and flexible environmental policy instruments provide enterprises with freedom to adjust environmental governance, resulting in significant cost pressures or economic incentives. The pressure of green technology innovation will be more directly applied to the decision-making level of enterprise management. Enterprises will choose to transform the external cost of pollution damage into the minimized internal control cost caused by technological innovation through the development of new technology, thus promoting the green technology innovation of enterprises. However, the green technology research and development activities of enterprises are often inseparable from the investment of internal capital. The objective existence of green technology R&D risk makes the investment return of R&D investment a huge uncertainty, and R&D activities usually indicate the strategic development direction of the enterprise. Enterprises are often reluctant to disclose specific information to the fund lender. Therefore, compared with tangible assets, it is difficult to obtain external financing with more stringent conditions. In addition, companies in green technology research and development based on the marginal cost of capital, usually give priority to internal funding. The pressure of taxes and transaction costs increases the internal capital burden of enterprises, which to some extent occupies the funds for green technology research and development, resulting in the decline of enterprise productivity. Therefore, this paper proposes research the hypothesis 2: the impact of market-incentive environmental policy instruments may promote or inhibit enterprise green technology innovation, so that the final effect is uncertain.

Finally, social-will environmental policy instruments often use the publicity of the news media to inform the public about environmental issues in construction projects. The public has always been a strong participant in environmental policy, mainly in the field of environmental impact assessment. There are two main ways for tools to contribute to corporate green technology innovation: first, through public disclosure through news media, enterprises will face pressure from public opinion to force enterprises to carry out green technology innovation; Second, through environmental news reports, the publicity effect of environmental protection awareness should be formed among the public, and enterprise managers should be encouraged to implement voluntary green technology innovation plans to gain good business image and other additional benefits in the public’s mind. Therefore, this paper proposes to study hypothesis 3: social-will environmental policy instruments can promote enterprises’ green technology innovation.

In summary, various types of environmental policy instruments cooperate with each other in the actual process of green innovation and development to form a relatively complete environmental policy system. Finally, it should be emphasized that the green technology innovation of enterprises with the participation of environmental regulation policy instrument system is a process of mutual cooperation, phased implementation and continuous innovation between regulators and enterprises, so the impact effect is a long-term process.

## 4. Research Model Design

### 4.1. Model Construction

Different types of environmental policy instruments have different impact mechanisms on green technology innovation, and their final impact effects may be different. To test the heterogeneous impact of different types of environmental policy instruments on green technology innovation, the panel data baseline regression model (1) was established as follows:

(1) $$gti_{it}=\alpha +\beta_{1}cac_{it}+\beta_{2}mi_{it}+\beta_{3}sw_{it}+\theta_{1}pgdp_{it}+\theta_{2}ihc_{it}+\theta_{3}ind_{it}+\varepsilon_{i}{}_{t}$$

where *i* denotes provinces and *t* denotes years, *gti* represents green technology innovation, *cac* represents command-control environmental policy instruments, *mi* represents market-incentive environmental policy instruments, and *sw* represents social-will environmental policy instruments. *pgdp*, *ihc* and *ind* represent three control variables respectively: level of economic development, innovative human capital and degree of industrialization. In addition, *α* is a constant term, *β*_1_, *β*_2_, *β*_3_, *θ*_1_, *θ*_2_ and *θ*_3_ represent the regression coefficient, and *ε* represents the random error.

Secondly, in order to further investigate whether there is a non-linear relationship between environmental policy instruments and green technology innovation, the panel threshold model is introduced for analysis. By referring to Hansen’s setting of panel threshold model, three kinds of conditions including three-threshold (model 2), double-threshold (model 3) and single-threshold (model 4) were investigated [44], and the modeling process from complex to simple was followed. Model design is as follows:

(2) $$\begin{array}{l} gti_{it}=\alpha_{i}+\beta_{1}EP_{it}l(q_{it}\leq \gamma_{1})+\beta_{2}EP_{it}l(\gamma_{1}<q_{it}\leq \gamma_{2})+\\ \;\;\;\;\;\;\;\;\;\;\;\;\;\;\;\;\;\;\;\;\;\;\;\;\;\;\;\;\;\;\beta_{3}EP_{it}l(\gamma_{2}<q_{it}\leq \gamma_{3})+\beta_{4}EP_{it}l(\gamma_{3}<q_{it})+\theta Z_{it}+\varepsilon_{it} \end{array}$$

(3) $$\begin{array}{l} gti_{it}=\alpha_{i}+\beta_{1}EP_{it}l(q_{it}\leq \gamma_{1})+\beta_{2}EP_{it}l(\gamma_{1}<q_{it}\leq \gamma_{2})+\\ \;\;\;\;\;\;\;\;\;\;\;\;\;\;\;\;\;\;\;\;\;\;\;\;\;\;\;\;\;\;\beta_{3}EP_{it}l(\gamma_{2}<q_{it})+\theta Z_{it}+\varepsilon_{it} \end{array}$$

(4) $$gti_{it}=\alpha_{i}+\beta_{1}EP_{it}l(q_{it}\leq \gamma )+\beta_{2}EP_{it}l(q_{it}>\gamma )+\theta Z_{it}+\varepsilon_{it}$$

Among them, threshold value: *γ*_1_ *< γ*_2_ *< γ*_3_, *gti* represents green technology innovation, *EP* represents a certain type of environmental policy tool, and *Z* represents the remaining explanatory variables, including the remaining environmental policy instruments and control variables. In addition, *l (·)* represents the indicator function, *q* is the threshold variable, *α_i_* is the intercept term, *β*_1_, *β*_2_, *β*_3_, *β*_4_ and *θ* represents the regression coefficient, and *ε* represents the random error.

There are two main tests for panel threshold model: one is to test whether there is threshold effect by constructing the corresponding likelihood ratio test statistic. The second is to test whether the threshold value is correctly selected. Taking the single threshold test (model 4) as an example, since the threshold value *γ* was unknown in this study, the value *γ*_0_ was selected from the threshold variable q to replace *γ*, so that the parameter estimators of the squared and minimized the γ_0_ were ${\hat{\beta }}_{1}$ and ${\hat{\beta }}_{2}$, and the null hypothesis of the threshold effect test was $H_{0}:\;\;{\hat{\beta }}_{1j}={\hat{\beta }}_{2j}$ (*j* was the number of explanatory variables), that is, there was no threshold effect. The null assumption for the threshold test was $\;H_{0}:\;\;\gamma =\gamma_{0}$, that is, the threshold value was correctly selected.

Finally, in order to reveal the difference in the impact effects of different types of environmental policy instruments on green technology innovation, with fixed control variable as base, the model of variable intercept of individual fixed effects and the variable coefficient of environmental policy with individual changes is used for analysis. The model is as follows:

(5) $$gti_{it}=\alpha_{i}+\beta_{i}EP_{it}+\theta Z_{it}+\varepsilon_{i}{}_{t}$$

where *gti* represents green technology innovation, *EP* represents a certain type of environmental policy instruments, and *Z* represents the remaining explanatory variables, including the remaining environmental policy instruments and control variables. In addition, *α_i_* is the intercept term, *β*_1_ and *θ* represents the regression coefficient, and *ε* represents the random error.

### 4.2. Variable Selection

The selection results of all variable indexes are shown in Table 1. The details are as follows:

(1) Explained variables

In the selection of indicators for explained variables, patents are exclusive, and enterprises can obtain profits from patents to compensate for R&D investment, and patents can effectively promote the innovation process (Ziedonis and Hall [45]). Therefore, the academic community widely uses the number of patents as an indicator of the level of technological innovation. The number of patents can be divided into the number of patent applications and the amount of patent authorizations. During the process of patent application, the enterprise has been influenced in internal technological innovation, production and operation, decision-making direction, etc. Moreover, the number of patent applications could be used to track the formation and development of new technologies. However, the amount of patent authorization is limited by the review procedure, which could result in the Inconsistency of time in reflecting green technology innovation activities. Therefore, when measuring corporate green technology innovation, this paper chooses the number of green technology patent applications as a measure of the green technology innovation level.

(2) Explanatory variables

① Command-control environmental policy instruments. The “three simultaneous” system is a strict supervision requirement and work system in the field of environmental protection system in China, which requires that environmental protection facilities in construction projects must be designed, constructed and put into use at the same time with the main project. Therefore, this paper chooses “three simultaneous” environmental protection investment volume as the index of command-and-control environmental policy instrument.

② Market-incentive environmental policy instruments. In recent years, sewage charges, as the most widely used market-incentive environmental policy, have gradually become effective in promoting pollution control and emission reduction. Therefore, this paper chooses the average amount of sewage charges paid by the enterprises that pay the sewage charges as the indicator of market-incentive environmental policy instrument.

③ Social-will environmental policy instruments. The reported environmental pollution and destruction events will generate public pressure on enterprises, so as to force enterprises to pay attention to environmental issues and carry out green technology innovation. Meanwhile, the news publicity of good environment also plays a positive role in guiding the green technology innovation of enterprises. Referring to the method of Jia and Zhao [46], the quantity of environmental information news disclosure is selected as an indicator to measure the social-will environmental policy instruments.

(3) Control variables

In terms of the selection of control variables, on the one hand, the level of economic development will affect the industrial reform and the overall financing situation, resulting in changes in the capital investment and related decisions of green technology innovation. On the other hand, with the increase of investment in innovative human capital, enterprises will become more productive and innovative, and pay more attention to the research and development of the emerging field of green technology innovation. Finally, at different stages of industrialization, enterprises attach different importance to the environment, thus affecting the demand for green technology. In summary, the control variables selected in this paper include the level of economic development, innovative human capital, and the degree of industrialization. Referring to the work of Pei et al. [47], we selected area per capita GDP, university or above proportion of employment and industrial added value and the ratio of GDP as a measure.

### 4.3. Data Source and Processing

In terms of data acquisition and processing of explained variables (*gti*), the number of green technology patent applications was obtained from the Patent Search and Analysis System of the Intellectual Property Office of China. The specific search methods are as follows: first, based on the classification of green technology in the China Green Patent Statistics Report (2014–2017) that published by the Planning and Development Department of the Intellectual Property Office of China in August 2018 (according to the *China Green Patent Statistics Report (2014–2017)*, green technologies mainly include alternative energy sources, environmental materials, energy conservation and emission reduction, pollution prevention and recycling technologies; source: www.sipo.gov.cn.). The the above classification is compared with the content of the IPC classification number in the IPC Green Inventory. Then, through the patent search and analysis system of the Intellectual Property Office of China, the corresponding patent data is retrieved according to the selected IPC classification number.

In terms of data acquisition and processing of core explanatory variables, ① Command-and-control environmental policy instrument (*cac*). “three simultaneous” environmental protection investment volume in construction projects comes from the annual China Environmental Yearbook(www.epsnet.com.cn). ② Market-incentive environmental policy instrument(*mi*). the sewage charge and the number of enterprises that pay sewage charge are from the annual China Environmental Yearbook(www.epsnet.com.cn). ③ Social-will environmental policy instruments (*sw*). The data of environmental information disclosure comes from the Duxiu academic retrieval system (www.duxiu.com). The method is to retrieve the number of environmental information disclosure in the most representative newspapers in 30 provinces of China.

In terms of data acquisition and processing of related indicators of control variables: ① Level of economic development (pgdp). Regional per GDP was obtained from China Statistical Yearbook (www.epsnet.com.cn). ② Innovative human capital (ihc). Numbers of employees with college degrees or above/total number of employees were obtained from China Science and Technology Statistical Yearbook (www.epsnet.com.cn). ③ Degree of industrialization (ind). Industrial added value/regional GDP, industrial added value and regional GDP were obtained from China Statistical Yearbook (www.epsnet.com.cn).

In addition, in this paper, all price-variable indicators are deflated with 2010 as the base period to eliminate the impact of price fluctuations on the results. A descriptive statistical analysis of the variable data is done (as shown in Table 2). It can be seen that the standard deviation of the green technology patent application amount is very large, indicating that there is a significant difference in the level of green technology innovation in 30 provinces in China. In addition, different environmental policy instruments also have large differences at the provincial level. In order to alleviate the influence of heteroscedasticity, the remaining variables except the two ratio-type indicators of innovative human capital and industrialization have been logarithmically processed in the regression analysis. And the correlation test is carried out, there is no correlation between three environmental policy instrument variables at the 1% level.

## 5. Empirical Results Analysis

### 5.1. Baseline Regression

A fixed-effect regression is done to model 1, and is verified by adding the control variables step by step (FE1, FE2, FE3 in Table 3) and adding the “heteroscedasticity-sequence correlation” robust standard error test (FE4 in Table 3). As can be seen from the regression results in Table 3, first, the command-control environmental policy instrument coefficients (ln*cac*) in the fixed-effect model are all positively significant at the confidence level of 10%. This means that command-control environmental policy instruments could promote green technology innovation, which is basically consistent with the theoretical hypothesis 1 proposed in Section 3, the possible reason is that, as a whole, in most of China’s 30 provinces and cities area, the implementation intensity of command-control environmental policy instrument has been maintained at a high level, and the negative effects of some provinces and cities maintained at a lower level have been ignored. This inference also requires subsequent panel threshold testing to prove it. Secondly, the market-incentive environmental policy instruments failed the significance test at the 10% confidence level, indicating that on the whole, market-incentive environmental policy instruments may have no impact on green technology innovation. This is consistent with theoretical hypothesis 2 proposed in Section 3. The possible reason is that, the positive and negative effects of the market-incentive environmental policy tools on the overall impact of the green technology innovations are mutually offset, the reason of insignificance may be that there is a nonlinear effect. This inference will be verified by subsequent panel threshold tests. In addition, the social-will type environmental policy instruments in the first two fixed effects regression coefficient model (FE0 and FE1) are positively significant at the 1% confidence level, indicating that social-will environmental policy instruments could promote green technology innovation, which is consistent with the theoretical hypothesis 3 proposed in Section 3, but did not pass the significance test at the 10% level in the latter three fixed effect models (FE2, FE3, FE4).

Besides, with the introduction of control variables, innovative human capital plays a significant positive role in promoting green technology innovation. In addition, in order to make the standard deviation between groups of the FE3 fixed effect model more robust, the “heteroscedasticity - sequence correlation” robust standard error test was added to correct the heteroscedasticity and sequence correlation problems. And it was found that the FE4 regression result didn’t change significantly from the previous several significant items, indicating that it passed the robustness test. Finally, according to the stability of the above conclusions, the role of command-and-control environmental policy instruments in promoting green technology innovation did not change due to the introduction of control variables. Moreover, in the regression results of FE4, the regression coefficient of command-control environmental policy instruments on green technology innovation is 0.1046 *, and the regression coefficient of market-incentive instruments is −0.093. Therefore, compared with market-incentive environmental policy instruments, command-control environmental policy instruments have a stronger effect on green technology innovation. The possible reasons for this conclusion are as follows: in recent years, the gradual development of Chinese government’s policy thinking on environmental protection. The implementation of the “three simultaneous” system of environmental protection earlier than the market incentives for sewage charges, and the system is more perfect, which leads to the rapid process of green technology innovation in different province.

### 5.2. Threshold Regression

**Figure 2:**
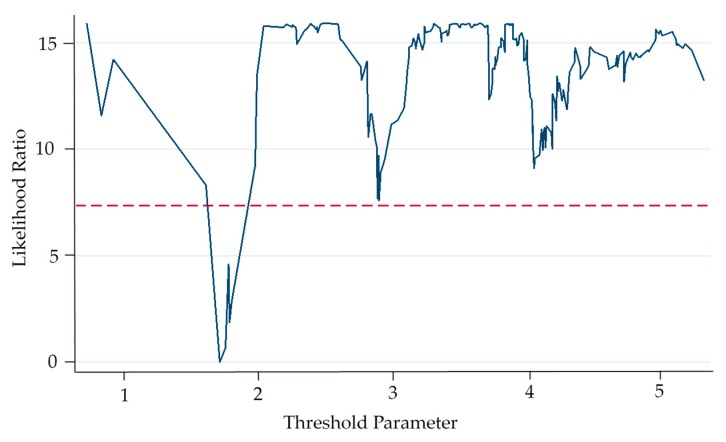
Single threshold regression LR test residue (ln*cac*).

Firstly, it can be seen from the results in Table 3 that the overall environmental policy instruments are not significant, probably due to the non-linear relationship between environmental policy tools and green technology innovation. Therefore, panel threshold regression is carried out, and the regression results are shown in Table 4. First, the three-threshold regression test was conducted on the command-control environmental policy instrument (ln*cac*), and the result showed that, the *p*-value of three-threshold was 0.5733, and the *p*-value of double-threshold was 0.2367. Therefore, accepting the null hypothesis, indicating that there was no three-threshold and double-threshold. And the *p*-value of single-threshold was 0.0400, thus rejecting the null hypothesis, indicating that there was a single threshold. Then, the single threshold test was carried out, and the single threshold *p* -value was 0.0433, rejecting the null hypothesis, indicating the existence of a single threshold and the threshold value *γ* = 1.6852, and the validity of the threshold was tested. As shown in Figure 2, the threshold value *γ* = 1.6852 falls within the effective estimation interval, that is, the null hypothesis is accepted, proving that likelihood ratio statistic—LR threshold value is correctly selected. In addition, due to logarithmic processing of variables before regression, the actual value converted into an index is 539.35 million yuan. It shows that when the investment of “three simultaneous” environmental protection projects is less than 539.35 million yuan (at which time the regression coefficient is −0.4386 **), command-control environmental policy instruments could inhibit the green technology innovation.

The above conclusion verifies the theoretical hypothesis 1. When the government adopts a loose command-control environmental policy instrument, the enterprises extracts some of the green technology R&D investment for pollution control in pursuit of excess profits. In the long term, when this kind of passive measures fail to achieve the desired effect of the pollution treatment, the enterprises can only reduce green technology R&D investment to compensate for environmental damage cost so as to avoid partial loss in excess profit, thus forming a vicious circle. This, in turn, when the command controlling environmental policy tool didn’t cross a “threshold” to maintain low control strength, its inhibition of green technology innovation has a significant impact. In addition, by comparing the descriptive statistics of variables in Table 2, it can be found that, this threshold value (539.35 million yuan) is at a low level, far lower than the mean value (5.69 billion yuan), which also supports the result analysis of baseline regression. When the “three simultaneous” environmental protection investment of the construction project is higher than 539.35 million yuan (the regression coefficient is 0.0826 at this time), the command-control environmental policy instrument can promote the green technology innovation, but the effect is not significant. The possible reason for the insignificant impact of this paper is: the regulation intensity is maintained above the threshold value, and with the increase of the command-control environmental policy supervision, some high-pollution and high-energy enterprises are forced to rectify or be forced to withdraw from the market. Green technology innovation is facing a lockout, so that the effect of command-and-control environmental policy tools on corporate green technology innovation is not significant above the threshold.

**Figure 3:**
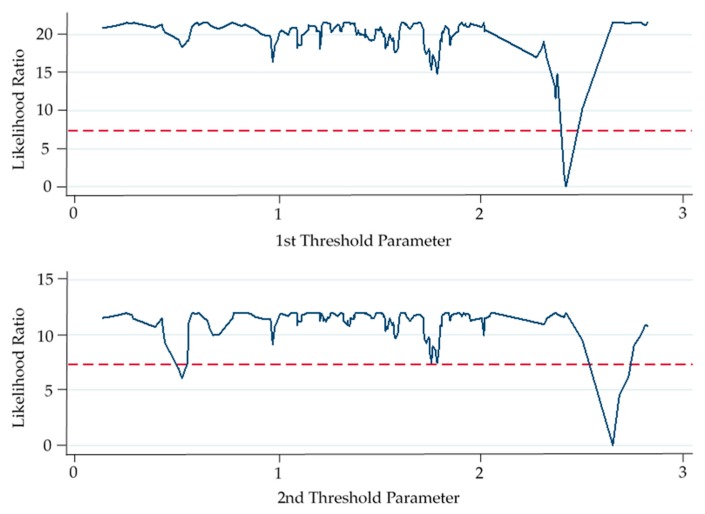
Double threshold regression LR test residuals (ln*mi*).

Secondly, a three-threshold regression test is conducted for market-incentive environmental policy instruments (ln*mi*). The three-threshold *p*-value is 0.1833, which means that the null hypothesis is accepted, indicating that there are no three thresholds. The two-threshold *p*-value is 0.0233, rejecting the null hypothesis, indicating that there are two thresholds. The *p*-value of single threshold is 0.3800, accepting the null hypothesis, that is, there is no single threshold. Therefore, the double-threshold analysis was conducted again, and it was concluded that the *p*-values of the double-threshold and the *p*-values of the single-threshold were 0.0100 and 0.3900 respectively, indicating that only the double-threshold existed with threshold values *γ*_1_ = 2.4153 and*γ*_2_ = 2.6469. Similarly, after conversion, the true value of the index are 111.93 thousand yuan and 141.10 thousand yuan respectively. In addition, the validity of the threshold value is tested. As shown in Figure 3, the threshold γ1 and γ2 both fall within the effective estimation interval, that is, the null hypothesis is accepted, and the LR test double threshold value is correctly selected.

The panel threshold regression results show that when the average amount of sewage charges paid by the enterprises that pay the sewage charges is lower than 111.93 thousand yuan or higher than 141.10 thousand yuan (regression coefficients are −0.1052 and −0.1087, respectively in Table 4), market-incentive environmental policy instruments could inhibit the green technology innovation, but the effect is not significant. When the amount is between the two (the regression coefficient is 0.2432), market-incentive environmental policy instruments have a positive impact on the green technology innovation, but the impact effect is not significant. This conclusion is consistent with hypothesis 2. The possible reason for this phased feature is that, when the regulatory intensity of market-incentive environmental policy instruments is maintained at a low level, the cost of pollution damage faced by enterprises does not exceed the return on investment brought by green technology R&D investment, in the short term, pollutant emissions have not been significantly reduced, leading to negative influence of market-incentive environmental policy instruments on corporate green technology innovation. In addition, excessive sewage charges may inhibit the development of the entire high-pollution and high-energy industry, causing most of its enterprises to lose their core competitiveness. Enterprises are difficult to survive, and green technology R&D will also face a lockout. Therefore, it can be found that excessively low or excessively high market-incentive environmental policy instruments may have negative impacts on corporate green technology innovation. However, in recent years, some enterprises have doubts and disputes about collecting fines for sewage charges. In view of this phenomenon, China’s environmental protection department has adjusted the accounting method of reasonable collection of sewage charges for many times. Therefore, the conclusion that market-incentive environmental policy instrument have double-threshold for green technology innovation has practical basis.

Finally, a three-threshold regression test was conducted for the social-will environmental policy tool (ln*si*). It was found that the *p*-values of three threshold, double threshold and single threshold were 0.3233, 1.0000 and 0.3800 respectively. Therefore, the null hypothesis was accepted, and demonstrating that there is no threshold effect. 

### 5.3. Regional Heterogeneity Analysis

A provincial fixed-effect regression analysis is done to model (5). The regression results are shown in Table 5. It can be seen that the command-control environmental policy instruments can effectively promote green technology innovation of the province in nine provinces, including Qinghai, Heilongjiang, Hubei, Fujian, Hainan, Guangxi, Hunan, Beijing and Zhejiang; the market-incentive environmental policy instruments have significantly promoted the green technology innovation in six provinces, including Shaanxi, Anhui, Xinjiang, Guizhou, Ningxia and Beijing; the social-will environmental policy instruments have effectively promoted the green technology innovation in 11 provinces, including Heilongjiang, Guizhou, Inner Mongolia, Jiangxi, Ningxia, Hebei, Tianjin, Anhui, Hubei, Shandong and Henan. On the whole, the effective promotion of different types of environmental policy instruments on green technology innovation is quite different at the provincial level. This indicates that the implementation intensity of different environmental policy instruments varies among provinces, and the leading role of green technology innovation on enterprises is influenced by the provincial technology market environment and the environment of mass entrepreneurship and innovation.

From the perspective of sub-region, and according to the criteria for the division of economic regions by the National Bureau of Statistics of China, China is divided into four regions: the eastern, central, western, and northeastern regions (according to the National Bureau of Statistics of China, *The Method of Dividing the Central and Eastern Regions of the East and the West*, the eastern region includes Beijing, Tianjin, Hebei, Shanghai, Jiangsu, Zhejiang, Fujian, Shandong, Guangdong, and Hainan; the central region includes Shanxi, Anhui, Jiangxi, Henan, Hubei, and Hunan; the western region includes Inner Mongolia, Guangxi, Chongqing, Sichuan, Guizhou, Yunnan, Tibet, Shaanxi, Gansu, Qinghai, Ningxia, Xinjiang; the northeastern region includes Liaoning, Jilin, Heilongjiang). After a fixed-effect analysis to model 1, the results are shown in Table 6. It can be seen that: first, the green technology innovation in the eastern region is mainly affected by the command-control environmental regulation policy. The possible reason is that, in eastern region, the level of economic development is relatively high, and enterprises have the potential and ability to conduct research and development of green technologies. They can promptly adjust feedback when responding to the government’s mandatory environmental policy. Second, the impact of environmental regulation policies in central and western regions on green technology innovation is not significant. The possible reason is that the central and western provinces have insufficient coordination and control capabilities in terms of different types of environmental policy tools.

Finally, the green technology innovation in the northeastern region is affected by both order-controlled and market-incentive environmental regulation policies, and the regression coefficients are negative. The possible reason is that the green technology innovation benefits brought about by command-control and market-incentive environmental policy instruments cannot compensate for the economic losses caused by excessive control. For example, the northeastern region is rich in coal resources, and the intensity of market incentives such as carbon emission trading and air pollution discharge fees is too high, which may seriously affect the operation. In addition, it can be seen from Table 6 that the social willing environmental policy tools may have no impact on the green technology innovation of the four regional. The possible reason is that the public’s participation in environmental issues is generally not high, and it has not stimulated the impact on corporate green technology innovation.

## 6. Conclusions and Policy Implications

Some findings of this paper are as follows: 

Firstly, in general, China’s environmental policy instruments have not fully played a role in promoting green technology innovation.

Secondly, there is a threshold effect between different types of environmental policy instruments and green technology innovation. Among them, there is single threshold effect between command-control environmental policy instruments and green technology innovation. When the domestic demand within a reasonable range crosses a certain threshold, that is, the investment in environmental protection of the construction project “three simultaneous” is higher than 539.35 million yuan, it is possible to lead to innovation of green technology; there is a double threshold effect between market-incentive environmental policy instruments and green technology innovation. Only when the intensity of market-incentive environmental policy instruments is maintained within a reasonable range, that is, the average amount of sewage charges paid by the enterprises that pay the sewage charges is between 111.93 thousand yuan and 141.10 thousand yuan, can the innovation of green technology be promoted. In addition, there is no obvious threshold effect between social will environmental policy instruments and green technology innovation.

Thirdly, among the control variables, innovative human capital could promote green technology innovation, while regional per GDP and degree of industrialization have no significant effect on green technology innovation.

Fourthly, the effects of different environmental policy instruments on green technology innovation vary greatly among provinces. Among them, there are nine provinces in which command-control environmental policy instruments can significantly promote green technology innovation; there are six provinces in which market-incentive environmental policy instruments can significantly promote green technology innovation; and in 11 provinces, social-will environmental policy instruments can significantly promote green technology innovation.

Fifthly, from the perspective of regions, green technology innovation in the eastern region is mainly influenced by command-control environmental policy instruments. The green technology innovation in northeastern region is mainly influenced negatively by command-control and market-incentive environmental policy instruments. Different types of environmental policy instruments in central and western regions may have no impact on green technology innovation. Moreover, the social-will environmental policy tools may have no impact on the green technology innovation of the four region.

The policy implications of this paper are as follows: firstly, command-control environmental policy instruments should be further developed to promote green technology innovation. Secondly, in terms of market-incentive environmental policy instruments, after the “sewage charges” are changed to “environmental taxes” in China, the relevant taxation standards should be improved to control the collection intensity within a reasonable range. In addition, the popularization of public ecological education should be formed, and the participation of the whole society in environmental issues should be discussed to solve the problem that social willing environmental policy tools have no significant impact on corporate green technology innovation. Thirdly, strict command -control environmental policy should be implemented in the eastern region, and relatively loose command-control and market-incentive environmental policy instruments should be implemented in the northeast region. Finally, the regional enterprises should strengthen the service guarantee of innovative talents management and actively absorb innovative talents to realize the effective promotion of green technology innovation.

## 7. Discussion

The research in this paper mainly answers three questions: First, are there heterogeneity effects of different environmental policy instruments on green technology innovation in China? Second, does the impact of different environmental policy tools on corporate green technology innovation have a threshold effect in China? Third, does the impact of different types of environmental policy tools on corporate green technology innovation have significant spatial differences in different regions of China? The main contribution of this paper is to obtain the green technology patent data manually to measure the green technology innovation level, to distinguish green technology patents from general technology patents, and to comprehensively investigate the heterogeneity of the impact effects of different types of environmental policy instruments on the green technology innovation.

This paper also has some shortcomings. Due to a lack of a unified measurement indicators of environmental regulation policies, this paper only selects representative indicators for three environmental policy instruments, and does not pay enough attention to the systematization of environmental policies and resources. In addition, in various regions, due to the different levels of synergy between different environmental policy instruments and green technology innovations, it may have an impact on the results. And some patent development strategy might had a disproportionate effect in a given year, this could affect the trajectory and rate of the response variable enormously, so it will be a direction of further research. Besides, more representative indicators and more comprehensive indicators are selected, which is also the focus of our research later. And the regional heterogeneity could also be attributed to difference in other policy instruments. Furthermore, there could be differences in enforcement etc. These are also possible reasons influencing the research results, which need further case study or empirical analysis in the future.

## Figures and Tables

**Table 1:** The selection results of variable indexes.

Type	Variable	Name	Unit	Index Selection
Explained variable	*gti*	Green technology innovation	pcs	Number of green technology patent applications
Explanatory variables	*cac*	Command-and-control	One hundred million yuan	Amount of construction project “three simultaneous” environmental protection investment
	*mi*	Market-incentive	Ten thousand yuan/units	Sewage charge/number of enterprises that pay sewage charge
	*sw*	Social-will	pcs	The number of environmental information disclosure news
Control variable	*pgdp*	Level of economic development	Per ten thousand yuan	Regional per GDP
	*ihc*	Innovative human capital	%	Number of employees with college degree or above/total number of employees
	*ind*	Degree of industrialization	%	Industrial added value/regional GDP

**Table 2:** Descriptive Statistical Results of Variables.

Variable	Unit	Obs	Mean	SD	Min	Max
*gti*	pcs	240	1011.45	1107.007	12	7581
*cac*	One hundred million yuan	240	56.8051	50.5471	0.3183	277.3061
*mi*	Ten thousand yuan/units	240	5.4372	3.4418	1.0649	18.9067
*sw*	pcs	240	44.7417	32.5459	4	279
*pgdp*	Per ten thousand yuan	240	3.4628	1.6253	1.3119	7.8679
*ihc*	%	240	16.9226	9.3121	6.4893	55.87
*ind*	%	240	38.5473	8.5236	11.8381	53.0361

**Table 3:** The results of fixed effect regression model of environmental policy instruments on green technology innovation.

Variable	FE0	FE1	FE2	FE3	FE4
ln*cac*	0.1010 * (2.14)	0.0993 * (2.10)	0.1045 * (2.33)	0.1046 * (2.33)	0.1046 * (2.16)
ln*mi*	−0.0473 (−0.47)	−0.042 (−0.41)	−0.0922 (−0.95)	−0.093 (−0.95)	−0.093 (−0.94)
ln*sw*	0.2017 *** (3.75)	0.2058 *** (3.76)	0.0809 (1.40)	0.0821 (1.37)	0.0821 (1.21)
ln*pgdp*		0.2489 (0.46)	0.5082 (0.98)	0.4794 (0.74)	0.4794 (0.63)
*ihc*			0.0436 *** (4.94)	0.0442 *** (3.81)	0.0442 ** (3.06)
*ind*				0.0008 (0.08)	0.0008 (0.06)
Obs	240	240	240	240	240
Prob > F	0.0009	0.0023	0.0000	0.0000	0.0002
AIC	211.4222	213.1794	188.2114	190.2047	188.2047
BIC	225.3447	230.5826	209.0952	214.5691	209.0885

Note: *** *p* < 0.01, ** *p* < 0.05, * *p* < 0.1. The values of *t* are in parentheses. The data in the table is compiled by Stata15.

**Table 4:** Regression results of threshold effect of different types of environmental policy instruments and green technology innovation.

Threshold Variable		Threshold Range	Regression Coefficient
ln*cac*	Three-threshold, double-threshold	null	
	Single-threshold	ln*cac* ≤ 1.6852	−0.4386 ** (−2.86)
		ln*cac* > 1.6852	0.0826 (1.88)
ln*mi*	Three-threshold	null	
	Double-threshold	ln*mi* ≤ 2.4153	−0.1052 (−0.92)
		2.4153 < ln*mi* ≤ 2.6469	0.2432 (1.59)
		ln*mi* > 2.6469	−0.1087 (−1.08)
ln*sw*	the threshold effect test failed		

Note: ** *p* < 0.05. The values of *t* are in parentheses. The data in the table is compiled by Stata15.

**Table 5:** Different types of environmental policy instruments affect the provincial estimation results of green technology innovation.

	ln*cac*		ln*mi*		ln*sw*
Qinghai	1.2498 *** (13.03)	Shaanxi	3.0620 ** (3.41)	Heilongjiang	1.8449 *** (3.96)
Heilongjiang	0.4776 ** (3.52)	Anhui	2.0432 *** (4.14)	Guizhou	1.3829 *** (4.05)
Hubei	0.3680 * (2.75)	Xinjiang	1.9779 *** (10.46)	Inner Mongolia	1.0211 *** (5.55)
Fujian	0.2684 *** (6.03)	Guizhou	1.8423 *** (12.50)	Jiangxi	0.5555 *** (5.62)
Hainan	0.2108 *** (9.97)	Ningxia	0.7098 *** (12.81)	Ningxia	0.4065 *** (7.08)
Shandong	0.1963 (1.19)	Yunnan	0.674 (1.86)	Hebei	0.3501 *** (8.14)
Guangxi	0.1760 *** (3.78)	Liaoning	0.5002 (0.43)	Tianjin	0.2850 * (2.72)
Hunan	0.1616 *** (25.18)	Beijing	0.1660 *** (5.55)	Anhui	0.2663 *** (7.26)
Beijing	0.1514 *** (3.97)	Chongqing	0.1539 (0.35)	Gansu	0.2652 (1.08)
Zhejiang	0.1396 * (2.43)	Hebei	0.1052 (0.48)	Hubei	0.2600 ** (2.79)
Chongqing	0.1096 (1.21)	Guangdong	−0.0717 (−0.16)	Shaanxi	0.2332 (1.85)
Sichuan	0.1063 (0.57)	Jilin	−0.1200 *** (−5.00)	Shandong	0.2178 * (2.59)
Gansu	0.0942 (1.56)	Hainan	−0.2053 *** (−4.22)	Jilin	0.1285 (1.93)
Guangdong	−0.068 (−0.57)	Sichuan	−0.2123 (−0.60)	Guangxi	0.1167 (1.34)
Hebei	−0.0766 (−1.75)	Henan	−0.2256 (−0.79)	Sichuan	0.0763 (0.87)
Tianjin	−0.0835 * (−2.19)	Tianjin	−0.2818 (−1.67)	Henan	0.0736 ** (3.44)
Jilin	−0.087 (−1.63)	Shanxi	−0.2857 (−0.95)	Zhejiang	−0.0015 (−0.02)
Jiangsu	−0.1228 (−0.69)	Hubei	−0.4450 *** (−4.30)	Chongqing	−0.0018 (−0.04)
Xinjiang	−0.1512 *** (−11.49)	Qinghai	−0.4526 (−1.10)	Jiangsu	−0.0065 (−0.03)
Shanghai	−0.1744 (−1.99)	Shanghai	−0.5928 ** (−2.86)	Xinjiang	−0.0125 (−0.37)
Liaoning	−0.2041 *** (−3.78)	Shandong	−0.7527 ** (−2.81)	Beijing	−0.1075 (−0.93)
Shanxi	−0.2061 (−1.96)	Gansu	−0.7601 *** (−5.44)	Yunnan	−0.1806 ** (−3.23)
Jiangxi	−0.2217 * (−2.06)	Zhejiang	−0.7637 (−1.37)	Liaoning	−0.191 (−2.00)
Shaanxi	−0.3182 *** (−4.70)	Hunan	−1.0177 *** (−9.48)	Shanghai	−0.2193 (−1.24)
Anhui	−0.3274 *** (−4.36)	Jiangxi	−1.0413 *** (−5.48)	Hunan	−0.2262 *** (−3.88)
Guizhou	−0.3681 *** (−6.31)	Jiangsu	−1.2320 *** (−7.09)	Qinghai	−0.2378 (−1.92)
Yunnan	−0.5345 *** (−6.08)	Inner Mongolia	−1.292 (−1.17)	Guangdong	−0.3168 *** (−15.24)
Ningxia	−0.5611 *** (−9.99)	Guangxi	−1.3187 *** (−7.07)	Shanxi	−0.5500 *** (−4.21)
Henan	−1.3392 *** (−11.44)	Fujian	−1.9814 *** (−4.36)	Hainan	−0.6650 *** (−7.93)
Inner Mongolia	−1.5916 *** (−3.91)	Heilongjiang	−5.5555 *** (−5.08)	Fujian	−1.6225 *** (−40.84)

Note: *** *p* < 0.01, ** *p* < 0.05, * *p* < 0.1. The values of *t* are in parentheses. And the *t* value with the addition of “heteroscedasticity—sequence correlation” robust standard error test is shown in parentheses. The data in the table is compiled by Stata15.

**Table 6:** Sub-regional estimation results of the impact of different environmental policy instruments on green technology innovation.

	Eastern	Central	Western	Northeastern
ln*cac*	0.1373 *	0.0915	0.0706	−0.2782 **
	(2.84)	(0.86)	(0.76)	(−10.91)
ln*mi*	−0.0255	−0.3566	0.1793	−0.0706 *
	(−0.18)	(−0.71)	(0.45)	(−4.47)
ln*sw*	−0.1215	0.1242	0.1479	0.0585
	(−0.81)	(1.00)	(1.07)	(0.78)
ln*pgdp*	−2.1679	1.8187	1.3746	2.0922 **
	(−1.93)	(0.70)	(1.08)	(12.01)
*ihc*	0.0598 *	0.0403	0.0456	−0.0414
	(2.57)	(0.78)	(1.27)	(−2.16)
*ind*	0.0233	0.0055	−0.0069	−0.0318 *
	(0.66)	(0.18)	(−0.29)	(−5.26)
Obs	80	48	88	24
Prob > F	0.0047	0.0053	0.0227	0.0452

Note: ** *p* < 0.05, * *p* < 0.1. The values of *t* are in parentheses. And the *t* value with the addition of “heteroscedasticity—sequence correlation” robust standard error test is shown in parentheses. The data in the table is compiled by Stata15.
